# GaN-Based Laser Wireless Power Transfer System

**DOI:** 10.3390/ma11010153

**Published:** 2018-01-17

**Authors:** Carlo De Santi, Matteo Meneghini, Alessandro Caria, Ezgi Dogmus, Malek Zegaoui, Farid Medjdoub, Boris Kalinic, Tiziana Cesca, Gaudenzio Meneghesso, Enrico Zanoni

**Affiliations:** 1 Department of Information Engineering, University of Padova, via Gradenigo 6B, 35131 Padova, Italy; matteo.meneghini@dei.unipd.it (M.M.); alessandro.caria@dei.unipd.it (A.C.); gaudenzio.meneghesso@dei.unipd.it (G.M.); enrico.zanoni@dei.unipd.it (E.Z.); 2 Centro Giorgio Levi Cases, University of Padova, via Marzolo 9, 35131 Padova, Italy; 3 Institut d’Electronique, de Microélectronique et de Nanotechnologie, Centre National de la Recherche Scientifique (IEMN-CNRS), Avenue Poincaré CS 60069, 59652 Villeneuve d’Ascq, France; ezgidogmus@gmail.com (E.D.); malek.zegaoui@iemn.univ-lille1.fr (M.Z.); farid.medjdoub@iemn.univ-lille1.fr (F.M.); 4 Department of Physics and Astronomy, University of Padova, via Marzolo 8, 35131 Padova, Italy; boris.kalinic@unipd.it (B.K.); tiziana.cesca@unipd.it (T.C.)

**Keywords:** laser diode, photodetector, wireless power transfer

## Abstract

The aim of this work is to present a potential application of gallium nitride-based optoelectronic devices. By using a laser diode and a photodetector, we designed and demonstrated a free-space compact and lightweight wireless power transfer system, whose efficiency is limited by the efficiency of the receiver. We analyzed the effect of the electrical load, temperature, partial absorption and optical excitation distribution on the efficiency, by identifying heating and band-filling as the most impactful processes. By comparing the final demonstrator with a commercial RF-based Qi system, we conclude that the efficiency is still low at close range, but is promising in medium to long range applications. Efficiency may not be a limiting factor, since this concept can enable entirely new possibilities and designs, especially relevant for space applications.

## 1. Introduction

Gallium nitride is an excellent material for electronic devices. Its high breakdown field, high energy gap and thermal stability make it suitable for high power circuits [1,2,3], and the high electron mobility ensures efficient high-frequency operation [3]. Additionally, it is a direct bandgap material, enabling optoelectronic devices such as LEDs, laser diodes and photodetectors, and the possibility of growing alloys containing aluminum and/or indium allows for the tuning of the wavelength from the UV-C to the green spectral range, widening its possible operating fields [4,5,6].

In this paper, we will present a new application of GaN-based optoelectronic devices. We designed a wireless power transfer system with no need for deployment of transmission channels or infrastructures, feature that proves useful in emergency conditions, such as for quick supply restoration after natural disasters or on difficult terrains, or for space operation. The system is based on a high power 405 nm laser diode, used to project a narrow energy beam over free space, and a high-periodicity multi-quantum well (MQW) photodetector, that converts the incoming photons into electrical power available for the load.

## 2. Experimental Details

**Figure 1:**
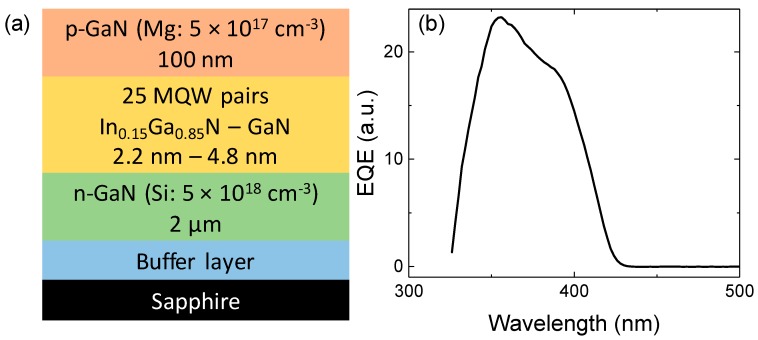
(**a**) Structure of the receiver and (**b**) photocurrent spectrum of a representative sample.

The core parts of the power transmission system, the source and the receiver, are fully GaN-based. The receiver is a 1 mm × 1 mm high periodicity MQW-based detector grown on c-plane single-side polished sapphire substrate by metal-organic chemical vapor deposition (MOCVD). Over an unintentionally doped GaN buffer layer, a 2 µm thick n-GaN:Si (5 × 10^18^ cm^−3^) is grown before the active region. The high periodicity MQW structure consists in 25 pairs of nominally undoped In_0.15_Ga_0.85_N/GaN quantum wells, 2.2 and 4.8 nm thick, respectively. The p-type layer is 100 nm thick and doped with Mg (5 × 10^17^ cm^−3^). A schematic sketch of the structure is reported in Figure 1a. The contact region is composed of a semi-transparent Ni/Au current spreading layer with Ni/Au grids. Additional details on device structure, growth and processing conditions, quality and performance can be found in [7]. The source is a high power 405 nm laser diode (nominal optical output power > 2 W at 1.5 A, manufacturer, city, state, country), chosen in order to excite the useful spectral range of the detector, shown in Figure 1b.

**Figure 2:**
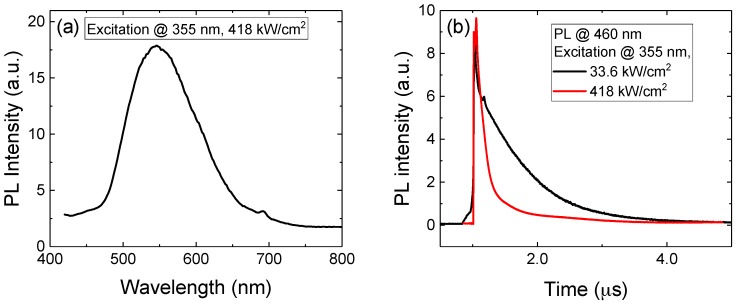
(**a**) Photoluminescence spectrum and (**b**) time-resolved photoluminescence decay at 460 nm of the epitaxial material of the receiver.

The external quantum efficiency of the receiver is about 17% at the 405 nm working wavelength [7], significantly limiting the performance of the whole system. In order to understand the origin of the losses, we carried out a characterization of the epitaxial material before processing. Photoluminescence (PL) measurements, both integrated and time-resolved, were performed exciting the samples with the third-harmonic (λ = 355 nm) of a 5 ns pulsed Nd:YAG laser (Brilliant, Quantel, Les Ulis, France) at a repetition-rate of 10 Hz. The luminescent emission was spectrally selected by a single grating monochromator (Oriel MS257, Newport Corporation, Irvine, CA, USA) and detected by a photomultiplier tube (R928, Hamamatsu, Hamamatsu City, Japan). A transient digitizer (TDS 7104, Tektronix, Beaverton, OR, USA) was used to record the PL signal evolution as a function of time to obtain the temporal decay curves. The photoluminescence (PL) spectrum, reported in Figure 2a, shows a significant emission from the deep levels responsible for the yellow luminescence band in GaN, supposed to be gallium vacancies (V_Ga_) [8], enhanced by the presence of impurities such as carbon [9,10,11] or oxygen [12,13]. The luminescence may originate from the quantum wells, suggesting a high defect density that may lower the efficiency due to defect-assisted recombination, or from the upper p-GaN layer, therefore leading to a reduction in efficiency caused by defect-mediated absorption of the transmitted power before the quantum wells. By analyzing the time-resolved decay of the PL at 460 nm, i.e., in a spectral region compatible with band-to-band recombination inside the quantum wells (see Figure 2b), it is possible to notice a long time constant that lowers at higher excitation levels, possibly caused by the low residual carrier density inside the quantum wells after the discussed defect-assisted absorption in the p-GaN layer.

**Figure 3:**
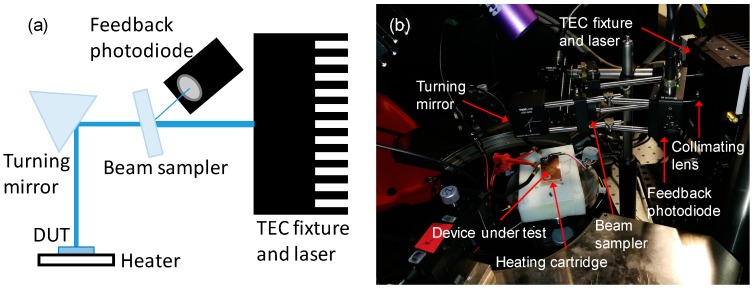
(**a**) Block diagram and (**b**) photo of the laser-based wireless power transfer system. All the main parts are highlighted.

The assembled system is shown in Figure 3. The laser diode is housed in a Peltier-cooled TCLDM9 high power mount connected to an ITC4005 combined laser diode and TEC controller. The mount also holds the C440TMD-A collimating lens. The laser beam is split by a BSF10-A UV fused silica beam sampler. A small part of the light is diverted to a PDA36A-EC photodetector, and a DG10-1500-A N-BK7 ground glass diffuser is used in order to reduce the optical power density on the photodiode, leading to improved performance and stability. The photodetector optical branch is used as a feedback system connected to the ITC4005 controller, ensuring that the optical power reaching the receiver is constant over time. The laser beam passing through the beam sampler is then directed to the receiver by a CM1-E02 dielectric coated turning prism mirror. The receiver is housed in a custom sample holder, mainly composed of a HT24S metal ceramic heater and a TH100PT 100 Ohm platinum resistance temperature detector connected to a TC200-EC temperature controller, used to stabilize the temperature of the device. The various optical power densities were obtained by changing the optical power setpoint in the feedback branch, i.e., by varying the bias current of the laser. For this reason, the size of the laser spot on the device under test is not constant and was measured for every setpoint. The power of the optical beam on the target plane was obtained for every setpoint by means of a calibrated S130VC photodiode power sensor and of a calibrated S142C integrated sphere photodiode power sensor, both connected to a PM100USB USB power meter interface. All the part numbers refer to products of the manufacturer Thorlabs (Newton, NJ, USA). The total length of the optical path, in this case, is 40 cm, but taking into account the good collimation and spatial coherence properties of a laser beam it can be easily increased.

## 3. Efficiency and Improvements

The overall efficiency of the power transfer system is strongly dependent on the wall-plug efficiency of the laser diode used in the system (in the present case 30%), which in recent reports is still slightly below 40% [14,15] but is expected to improve in the future due to growth and technology improvements. This value may significantly change with different models, manufacturer and specification sets. For this reason, in the following we will refer to the optical-to-electrical power conversion efficiency (η_OE_), defined as the ratio between the maximum electrical power provided by the receiver and the optical power incident on it. Given a specific laser diode model, the total efficiency may be obtained by multiplying η_OE_ and the wall plug efficiency of the laser. A second important parameter is the efficiency of the receiver, that is about 17% at the 405 nm working wavelength [7]. This value is relatively low since no specific strategy to improve the light coupling (e.g., surface roughening, dielectric mirrors, etc.) has been implemented in the present setup. Both GaN-based laser diodes and photodetectors were already shown to possess excellent reliability [14,16], ensuring that the system will not lose efficiency when operated for a long time.

### 3.1. Electrical Load

An important parameter for every optoelectronic receiver is the value of the equivalent load resistance, that needs to be designed in order to maximize the power transfer. In order to understand its impact, under every excitation power density and external condition reported in the following sections we submitted the MQW detector to a full load voltage sweep monitoring the output current variation by means of a HP 4155A semiconductor (Keysight, Santa Rosa, CA, USA) parameter analyzer.

**Figure 4:**
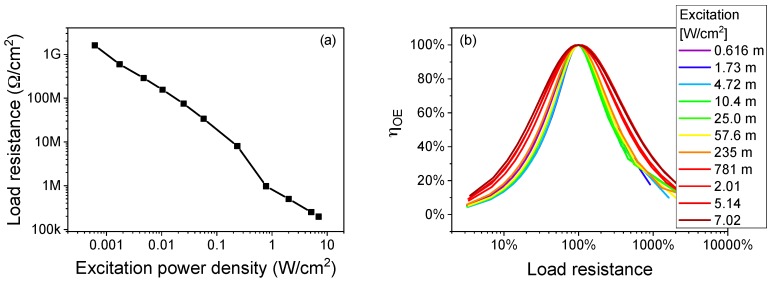
(**a**) Optimal load resistance value at different excitation power levels of the designed wireless power transfer system and (**b**) experimental variation in efficiency when the load resistance is varied with respect to the optimal one.

As can be seen in Figure 4a, the load resistance leading to the maximum efficiency decreases with increasing excitation level, due to the higher amount of current flowing through the receiver. When the load varies from the optimal one, as shown in Figure 4b, we detected a steep decrease in efficiency for lower resistance values, whereas the receiver is less sensitive to an increase in load resistance. The negative effect of the load change is mitigated by a higher excitation power density. This large variation implies that the operating condition of the receiver should be chosen in advance, to allow for design of the optimal equivalent load resistance. If the load value is chosen for low-power operation, the efficiency of the whole system would be significantly reduced when the excitation power level is high. In the following, efficiency always refers to the peak efficiency detected during the voltage sweep.

### 3.2. Dependence on Temperature

**Figure 5:**
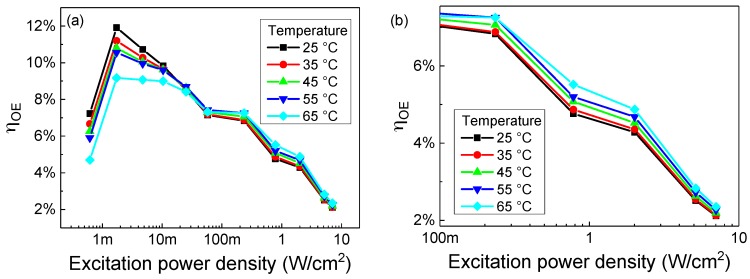
(**a**) Optical to electrical conversion efficiency of the designed laser-based wireless power transfer system as a function of the excitation power density at various receiver operating temperatures; The high-energy region is zoomed in (**b**).

The measured η_OE_ of the system is shown in Figure 5 as a function of the excitation power density and of the receiver temperature.

When temperature increases, at low excitation levels the efficiency decreases. This effect is compatible with the increase in Shockley-Read-Hall recombination rate inside the quantum wells of the receiver: at higher temperature a larger part of the photo-generated electron-hole pairs are captured and recombine in the defects, leading to a lower current collected at the contacts of the photodetector. The opposite trend is visible under high excitation: since the SRH recombination is a rate-limited process, once the generation is high enough and saturates the maximum capture rate at all the temperatures the efficiency does not decrease with temperature anymore. Instead, an increase in temperatures enhances the carrier escape from the quantum wells due to thermionic emission or phonon- and trap-assisted tunneling processes [17,18,19], causing a higher current (and therefore efficiency) at high temperature.

The behavior as a function of the power density can be explained through similar considerations. Initially, the efficiency increases due to the saturation of the SRH recombination centers. The decrease up to 50 mW/cm^2^ is likely related to the heating of the device under the higher power density, since its amount is lower at higher temperature and the effect is more intense the higher the power density. The second decrease at 200 mW/cm^2^ is not caused by heating, since temperature increases the efficiency, and may be related to the higher band filling at high excitation density, that reduces the percentage of absorbed incoming photons. This hypothesis is consistent with the lower efficiency improvement caused by the temperature at the two highest intensity levels: the almost complete filling of the quantum wells leads to a significant carrier escape from the quantum wells even at lower temperature.

In this case, the surface of the receiver is not optimized to reduce the reflection of the incoming light at the interface between the air and the device, which reduces the peak efficiency. Additional tests are currently in progress, by coating the surface of the detector with a material of intermediate refractive index to reduce the reflected component and by using nanostructured surfaces.

### 3.3. Partial Absorption

**Figure 6:**
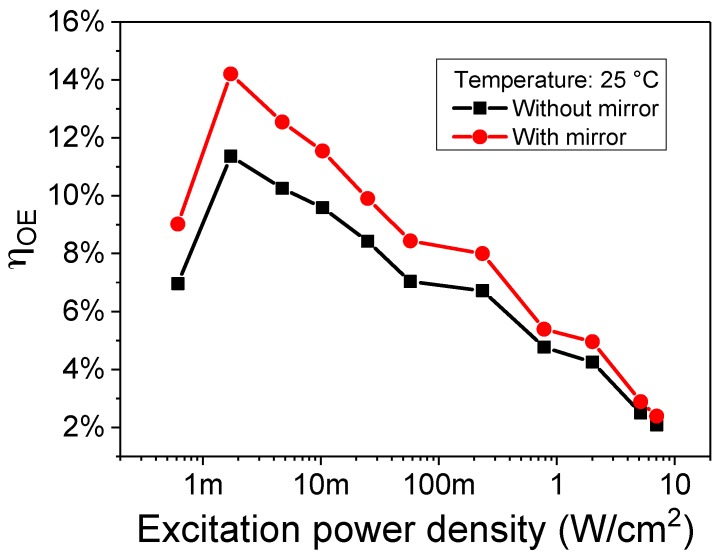
Variation in efficiency when a generic broadband mirror is placed below the receiver to compensate for partial absorption.

An additional loss channel in the system is the partial absorption of the incoming light. Even though the receiver has 25 QWs, part of the photons may pass through it without getting absorbed, leading to an efficiency reduction. In order to detect this possible process, we placed the detector over a Thorlabs PF10-03-P01P broadband silver mirror, with a nominal 92% reflectivity at 405 nm and at 12° angle of incidence, and carried out an additional characterization set, summarized in Figure 6. The presence of the mirror significantly increases the overall η_OE_ of the entire system above 14%, close to the maximum of 17% that is due to the limitation in the receiver efficiency. Even though the obtained system efficiency in this preliminary work is still low, it is comparable to early reports on other wireless power transfer techniques [20,21,22,23].

The result of this set of measurements may confirm the assumption, made in the previous section, that the second decrease in efficiency is caused by the band filling. As a matter of fact, the beneficial effect of the mirror is more prominent at low excitation power density, where a lot of states are available for the collection of the reflected light, and almost negligible at high intensity, due to the almost complete filling of all the states that may be able to collect the reflected photons.

A more advanced solution of the issue involves deposition of a suitable reflecting layer on the backside of the detector substrate, designed in order to have high performance and reflectivity at the operating wavelength, and is currently under development.

### 3.4. Laser Beam Uniformity

The last loss mechanism under analysis originates directly from the design of the system. Since the laser source is focused directly on the detector, the optical power density on its surface is significantly uneven. Therefore, all the dark areas act as a load for the illuminated region, and part of the photo-generated hole-electron pairs flow through them as forward current, reducing the efficiency. The solution we tested in this case is the use of a Thorlabs DG10-1500-A N-BK7 ground glass diffuser along the optical path, that leads to a uniform optical power density over the whole photodetector.

**Figure 7:**
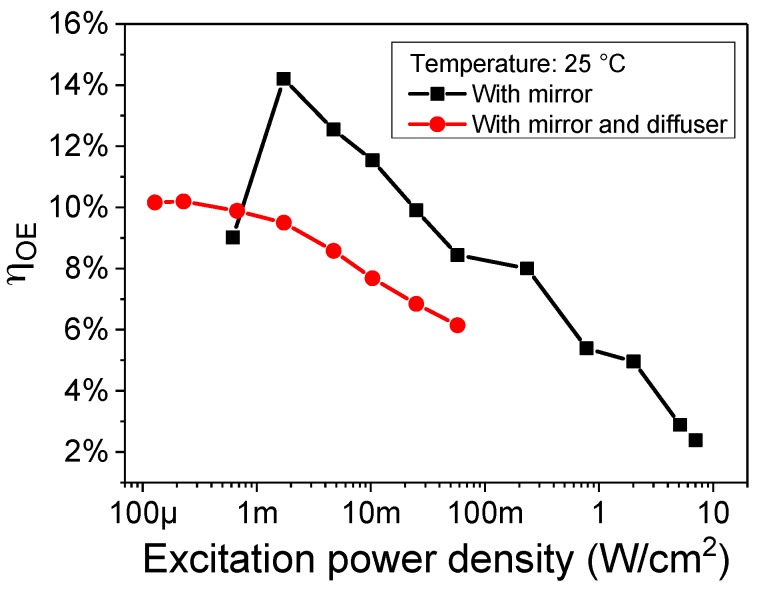
Variation in efficiency with a more uniform excitation over the whole receiver area.

Figure 7 reports the comparison between the η_OE_ of the system, including the mirror, with and without the diffuser. At low power densities, the improvement caused by the diffuser is evident. Remarkably, the trend is reversed above 1 mW/cm^2^, where the efficiency is higher without the diffuser. A possible explanation may be the higher heating of the receiver, since for the same excitation density a larger area is covered by the laser light and therefore the temperature of the receiver is higher. This may be confirmed by the extrapolation of the expected η_OE_ at very high power densities, that converges at the value of the system without diffuser but with more heating and band filling, but the root cause of this difference is still under investigation.

Even though the use of the diffuser leads to a lower efficiency, the amount of power transferred is about one order of magnitude higher with respect to the use of the sole mirror, therefore a diffuser at receiver side may be a viable option when the main goal is not the peak efficiency but the transfer of as much energy as possible in a short amount of time.

### 3.5. Comparison with Commercial Wireless Power Transfer System

**Figure 8:**
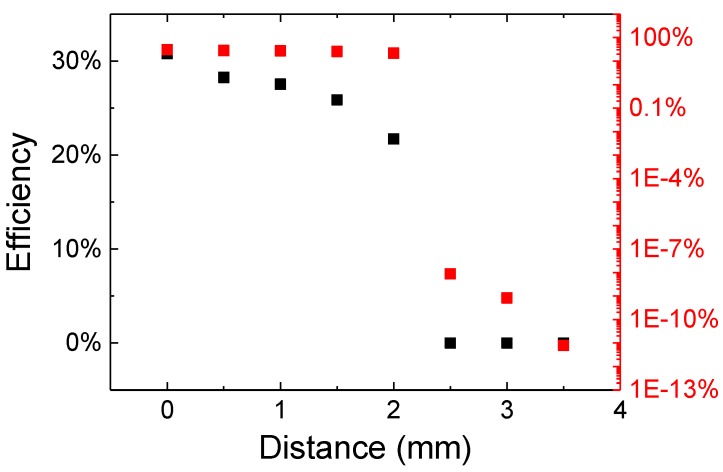
Efficiency, in linear and logarithmic scale, of a commercial Qi wireless power transfer system as a function of the distance between transmitter and receiver.

In order to understand the benefits of the designed system, we compared its performance with a commercial wireless power transfer system based on the Qi standard [24,25]. Its efficiency as a function of distance is reported in Figure 8, where distance 0 indicates the design working distance of the system, i.e., the when the transmitter and the receiver are in contact. As can be noticed, at this working distance the efficiency of the Qi system is above 30%, and drops rapidly to about 20% when the distance increases to 2 mm. Above this value, the receiver is no longer able to collect power for the load and the efficiency sharply drops, even though the Qi transfer protocol is still enabled and the receiver is powered. When the distance increases to 5 mm, the receiver powers down and the transfer completely stops.

More advanced designs include the four-coils design, which is able to achieve efficiency as high as 92.5% at 15 cm and 32.1% at 1 m [26], but are anyway limited by the typical decay of the efficiency with increasing distance of inductive wireless power transfer systems [27]. The transmission distance can be maximized according to the “maximum power transfer” theorem design rule, but this limits efficiency to 50% [25]. A good discussion on the efficiency and performance of the various approaches is given in [28]. Even though these solutions may have a good efficiency in the short range, for medium range operation the laser-based system is a better solution. Additionally, it is possible to switch between the “maximum power transfer” and the “maximum transfer efficiency” mode simply by changing the bias current of the excitation laser (see Figure 4), without any physical change to the transmitter or receiver architecture, needed in the case of different modes in RF systems.

## 4. Applications

One of the main advantages of the proposed power transfer system is its free-space operation mode, that does not rely on any pre-determined environmental condition or on the deployment of specific infrastructures. Therefore, since the transmission channel cannot be modified by the user in order to obtain the best performance, a detailed analysis of its behavior should be carried out.

**Figure 9:**
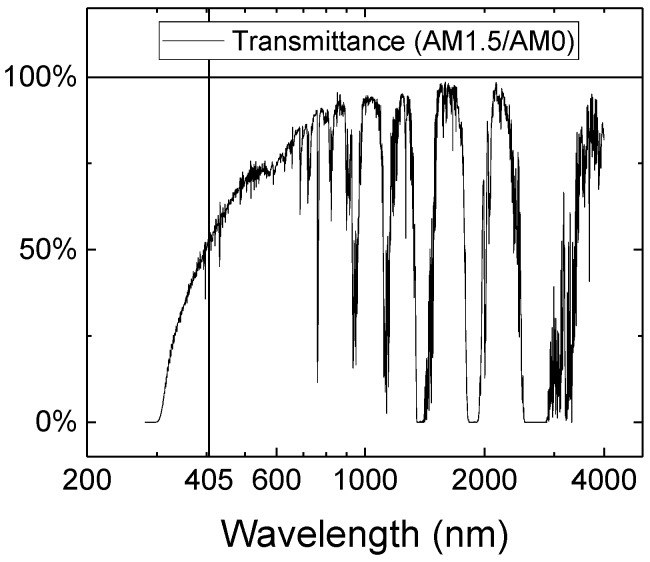
Air transmittance, computed as the ratio between the AM1.5 and the AM0 spectra.

For applications on earth’s surface, transmission takes place in air. The higher Rayleigh scattering cross-section at lower wavelengths disfavors straight-line beams in the blue spectral region [29], due to the higher scattering of photons in other directions. In the UV region, the strongest loss cause is the absorption by molecular oxygen and ozone, whereas the main absorption bands in the visible range are oxygen, ozone and water vapor [30]. The latter is the origin of the losses in the IR range, with some contributions by CO_2_ (in the 2.7 μm band), N_2_O, CO and CH_4_. Figure 9 shows the transmittance of the atmosphere, computed as the ratio between the Air Mass 1.5 (AM1.5) and Air Mass 0 (AM0) spectra according to the ASTM G173-03(2012) and ASTM E490-00a(2014) standards, respectively. As can be noticed, the 405 nm laser used in this work is not the best option for power transfer in air, especially if compared to systems working in the red/infrared range, as the laser-powered model aircraft recently demonstrated by NASA [31] or the systems designed by other groups [32,33]. Even though the transmittance is only about 50%, this value can be improved moving to royal blue or green lasers. This value is relative to the absorption of a column of atmosphere roughly 13.5 km thick, therefore the use of violet lasers may still be suitable for medium range transfer. The useful range of the laser-based approach is longer even compared to microwave wireless power transfer systems, which is usually in the range of tens or few hundreds of meters, and it is also favored by a more compact and light apparatus [23].

This discussion does not consider the different composition of the atmosphere at different altitudes [30], therefore more accurate measurements of air transmittance at surface level are needed. Additionally, the variation in efficiency of lasers and detectors operating in different spectral ranges, as well as their reliability, needs to be taken into account, leaving room for earth surface-level applications based even on blue laser, exploiting the good performance and robustness ensured by the wide-bandgap gallium nitride.

The use of the presented power transfer system at earth surface level is still debatable, but the performance loss caused by the scattering is no more an issue for space applications, where applications in the blue spectral range can take advantage of the superior thermal stability and radiation hardness of gallium nitride [34,35,36,37,38].

### 4.1. Inter-Satellite Power Trasfer Optimization

The current architecture for satellite powering is significantly un-optimized. Every satellite has its own set of solar panels, as well as mechanical components and circuitry for deployment and control. This yields an increase in the payload (and in the cost) for every space mission. Secondly, once the power supply system on a specific satellite fails, due to damage, wear-out or erroneous deployment, the entire system powers down without any back-up possibility.

The literature on satellite failure causes is complex and often shows only aggregated data. Robertson and Stoneking in an early report reviewed data on on-orbit anomalies and failures of satellites launched from 1990 to 2001 [39]. The Electrical Power System (EPS) subcategory, including solar array, battery, bearing and power transfer assembly, DC-DC converters and power regulators accounted for 21.1% of the total anomalies and 11.6% of the total critical failures. Rodiek and Brandhorst carried out a detailed analysis of the satellite solar array reliability in the years between 1998 and 2008 [40], and they report that power anomalies ranked the third cause for anomalies (at 19% of the total), solar array anomalies accounting for the majority of them. They gained further insight by analyzing the corresponding insurance claims, finding that power anomalies accounted for 47% of the total, and that solar arrays were the cause in 69% of the cases. Additionally, they show that, in the year 2004, solar arrays caused 49% of the value of insurance claims. Castet and Saleh showed that solar array operation and deployment account for 25% of the causes for early failure (30 days) of satellites, and contribute for a 12% in the long term (10 years), analyzing only failures of 1584 earth-orbiting satellites launched between January 1990 and October 2008 [41]. Recently Langer and Bouwmeester analyzed the failure causes of 178 CubeSats launched up to 30 June 2014 [42], showing that the aggregated failures caused by electrical power systems after 90 days account for 36% of the total failures, including dead-on-arrival.

**Figure 10:**
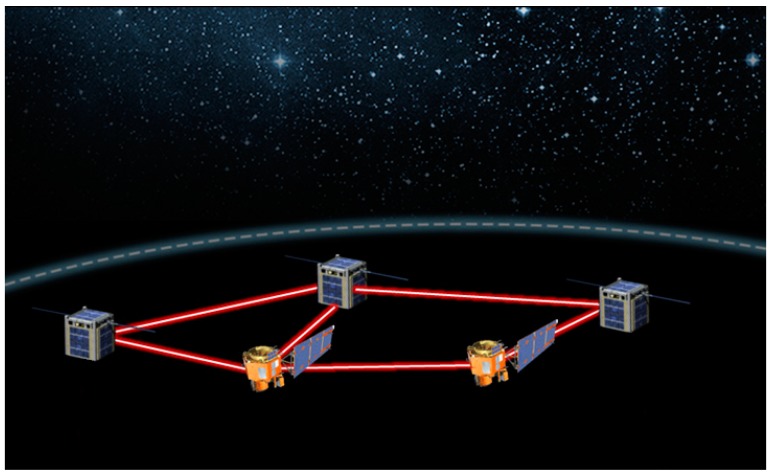
Sketch of an optimized satellite power generation and transfer system. Adapted from https://www.nasa.gov/sites/default/files/thumbnails/image/laser_comm_workshop_image.png.

If failures caused by solar arrays are such a relevant part, more advanced power distribution systems can be designed, taking advantage of the proposed wireless power transfer method. As sketched in Figure 10, a new power architecture may divide the satellites into two categories: producers and consumers. Producers are satellites designed for efficient power harvesting and equipped with lasers for conveying the power to the consumers. Consumers are the functional satellites, containing all the telecom or scientific equipment as well as receivers for the laser beams but no solar panels. One of the main advantages is the following: only the producers are equipped with solar arrays and corresponding deployment elements and circuitry, leading to a lower payload for consumers and therefore to a lower mission cost. Secondly, the system is more robust and allows for redundancy: if the solar harvesting fails on one producer, a second one may deliver power to the consumers, while an additional producer is built to replace the first one. Additionally, the producer and the consumer design constraint are reduced, and the producer structure and design can be completely changed to reflect its new role and boost the harvesting efficiency. This new architecture may also be used as redundancy or back-up plan in the current architecture, keeping the solar panels on all satellites but providing a second channel of power supply in case of failures.

### 4.2. Emergency Power Transfer

**Figure 11:**
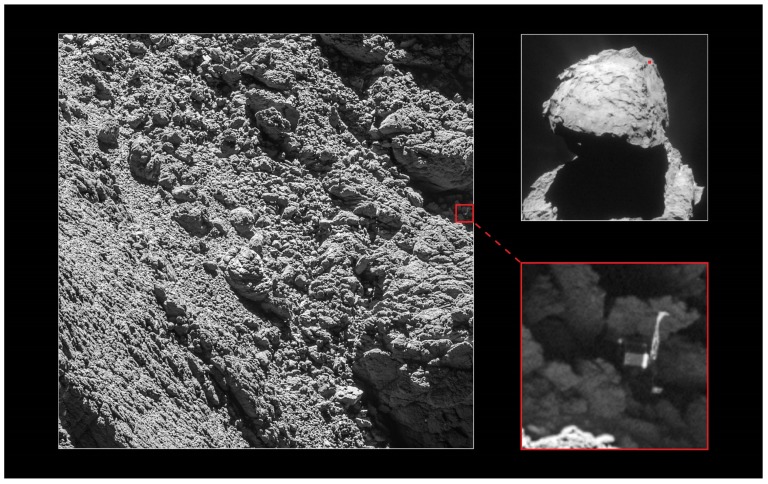
Photo of the Philae lander, stuck into a crack on comet 67P/Churyumov-Gerasimenko, revealed by the OSIRIS narrow-angle camera on the Rosetta orbiter. Retrieved from http://www.esa.int/var/esa/storage/images/esa_multimedia/images/2016/09/philae_found/16114811-1-eng-GB/Philae_found.jpg.

A second possible application of a laser-based power transfer system in space may be suggested by the outcome of a recent space mission. The European Space Agency (ESA) Rosetta space probe, launched on 2 March 2004, was designed to reach the comet 67P/Churyumov-Gerasimenko. After arrival on 6 August 2014, it started orbiting around the comet collecting data while launching the lander Philae on the comet surface. Unfortunately, after two days of operation the battery of the lander was drained, and communication between the lander and the orbiter was possible only for brief moments, limiting the amount of data provided by the mission. It was later discovered that the lander failed to hook on the comet surface at the designed location, ending up stuck in a dark part of the comet (see Figure 11). In this case, equipping the probe with an emergency power transfer composed of a laser on the orbiter and a receiver on the lander can allow for an additional failsafe battery recharge option. Even the structure of the lander may be changed, favoring more round or articulated shapes that may allow it to reposition after the emergency power transfer. If the total cost of a space mission is taken into account, such low-cost, low-weight emergency power transfer system may provide a significant added value.

## 5. Conclusions

In summary, within this work we demonstrated the feasibility of a gallium nitride-based wireless power transfer system, composed of a blue laser used as the source and of a suitable photodetector as the receiver. After optimization, the optical-to-electrical conversion efficiency exceeds 14%, and the main limiting factors is the peak EQE of the receiver, which can be improved through surface optimization and optimized quantum well design. Efficiency losses are found to originate mostly from heating and band-filling, which reduce the overall efficiency.

The goal of this work is not to report record efficiencies, but to present a new architecture that can enable new applications, designs and technologies. This system is a competitive alternative for medium to long range power transfer on earth without any need for the deployment of dedicated infrastructures, even compared to IR-based approaches, and has a lower weight and size. Additionally, it has no competitors for space applications, where the use of dedicated transfer channels, such as cables, is not possible, and may prove effective at least as an emergency power transfer method. The good thermal stability, robustness and radiation hardness of gallium nitride is an added value in all these cases.
